# Effects of Mlx-8, a phospholipase A_2_ from Brazilian coralsnake *Micrurus lemniscatus* venom, on muscarinic acetylcholine receptors in rat hippocampus

**DOI:** 10.1590/1678-9199-JVATITD-2019-0041

**Published:** 2020-01-27

**Authors:** Roberta Tancredi Francesco dos Santos, Marcelo Florencio Passos Silva, Rafael Marques Porto, Ivo Lebrun, Luís Roberto de Camargo Gonçalves, Isabel de Fátima Correia Batista, Maria Regina Lopes Sandoval, Fernando Maurício Francis Abdalla

**Affiliations:** 1 Laboratory of Pharmacology, Butantan Institute, São Paulo, SP, Brazil.; 2 Laboratory of Biochemistry and Biophysics, Butantan Institute, São Paulo, SP, Brazil.; 3 Laboratory of Pathophysiology, Butantan Institute, São Paulo, SP, Brazil.

**Keywords:** Muscarinic receptors, Hippocampus, Micrurus lemniscatus, Inositol phosphate, Phopholipase A_2_

## Abstract

**Background:**

Here, we described the presence of a neurotoxin with phospholipase A_2_ activity isolated from *Micrurus lemniscatus* venom (Mlx-8) with affinity for muscarinic acetylcholine receptors (mAChRs).

**Methods:**

The purification, molecular mass determination, partial amino acid sequencing, phospholipase A_2_ activity determination, inhibition of the binding of the selective muscarinic ligand [^3^H]QNB and inhibition of the total [^3^H]inositol phosphate accumulation in rat hippocampus of the Mlx-8 were determined.

**Results:**

Thirty-one fractions were collected from HPLC chromatography, and the Mlx-8 toxin was used in this work. The molecular mass of Mlx-8 is 13.628 Da. Edman degradation yielded the following sequence: NLYQFKNMIQCTNTRSWL-DFADYG-CYCGRGGSGT. The Mlx-8 had phospholipase A_2_ enzymatic activity. The pK_i_ values were determined for Mlx-8 toxin and the M_1_ selective muscarinic antagonist pirenzepine in hippocampus membranes via [^3^H]QNB competition binding assays. The pK_i_ values obtained from the analysis of Mlx-8 and pirenzepine displacement curves were 7.32 ± 0.15, n = 4 and 5.84 ± 0.18, n = 4, respectively. These results indicate that Mlx-8 has affinity for mAChRs. There was no effect on the inhibition ability of the [^3^H]QNB binding in hippocampus membranes when 1 µM Mlx-8 was incubated with 200 µM DEDA, an inhibitor of phospholipase A_2_. This suggests that the inhibition of the phospholipase A_2_ activity of the venom did not alter its ability to bind to displace [^3^H]QNB binding. In addition, the Mlx-8 toxin caused a blockade of 43.31 ± 8.86%, n = 3 and 97.42 ± 2.02%, n = 3 for 0.1 and 1 µM Mlx-8, respectively, on the total [^3^H]inositol phosphate content induced by 10 µM carbachol. This suggests that Mlx-8 inhibits the intracellular signaling pathway linked to activation of mAChRs in hippocampus.

**Conclusion:**

The results of the present work show, for the first time, that muscarinic receptors are also affected by the Mlx-8 toxin, a muscarinic ligand with phospholipase A_2_ characteristics, obtained from the venom of the Elapidae snake *Micrurus lemniscatus*, since this toxin was able to compete with muscarinic ligand [^3^H]QNB in hippocampus of rats. In addition, Mlx-8 also blocked the accumulation of total [^3^H]inositol phosphate induced by muscarinic agonist carbachol. Thus, Mlx-8 may be a new pharmacological tool for examining muscarinic cholinergic function.

## Background

In the Americas, the Elapidae family is represented by coralsnakes that comprise 120 species and subspecies belonging to the genera *Micruroides*, *Leptomicrurus* and *Micrurus* [1, 2]. *Micrurus* is the most abundant and diverse genus with many species found in South and Central America and the Southern United States [3-6]. However, the biochemistry and pharmacology of components from coralsnake venoms have not yet been thoroughly studied. 

Currently, *Micrurus lemniscatus* is a species composed of three subspecies (*M. l. carvalhoi*, *M. l. helleri* and *M. l. lemniscatus*). Particularly, *M. l. carvalhoi* is distributed along the Brazilian east coast from the northeast to southeast of the country and in parts of central, central-western, southeastern and southern Brazil, as well as eastern Paraguay and northeastern Argentina [7, 8]. Moreover, the venom of this animal is composed of approximately 70% three-finger toxins (3FTxs) and 10% phospholipase A_2_ (PLA_2_) toxins [9]. While enzymatic toxins contribute mainly to slow immobilization and digestion of prey, the non-enzymatic toxins stimulate rapid immobilization through their neurotoxic or cardiotoxic effects [10].

In the elapid envenomation the presynaptic neurotoxins or β-neurotoxins and postsynaptic neurotoxins or (-neurotoxins are recognized as major and most important components of these venoms [11-13]. β-neurotoxins are characterized by their PLA_2_ activity while (-neurotoxins can be characterized as 3FTx enzymatic-free proteins that interact with cholinergic nicotinic receptors and others that interact with muscarinic acetylcholine receptors (mAChRs).

Secreted PLA_2_, found in mammals and animal venoms, have a molecular weight between 12 and 19 kDa, have five to eight disulfide bridges and need millimolar calcium concentrations for its catalytic activity [14]. Among the main components of animal venoms are the secreted PLA₂ that belong to distinct PLA₂s groups. Snake venom PLA₂s from Elapidae and Viperidae families belong, respectively, to the IA and IIA/IIB groups [15, 16]. For instance, snake venoms are rich sources of PLA_2_ enzymes that are frequently found as a large number of isozymes [17]. 

Based in transcriptomic data it can be observed that *Micrurus* species are arranged in an approximately northwestern to southeastern sequence, the high PLA_2_ and low 3FTx concentrations in the North to high 3FTx and low PLA_2_ concentration in the South [9]. In this way, the proteomics of the *Micrurus* venoms present a great diversity concerning the PLA_2_ composition. *M. surinamensis and M. l. carvalhoi* venoms show relatively little PLA_2_ activity. However, activity does not necessarily reflect the amount of PLA_2_ present. Structure determination of new micrurine PLA_2_ illustrates their great structural diversity. Of 121 PLA_2_s with partial or complete structures, the majority are apparently catalytic, having the requisite H48, D49, Y52, and D101 in their active sites. The remains are apparently non-catalytic [see 9, for review].

Quantitative differences in the content of 3FTx and PLA_2_ might reflect directly in the pharmacological and biological activities of *Micrurus* venoms. On the other hand, Tanaka et al. [12] showed that *M. frontalis, M. ibiboboca and M. lemniscatus* venoms contain different levels of PLA_2_ activity, although the venom of *M. frontalis* seems to have a lower hydrolytic activity when compared to *M. lemniscatus and M. ibiboboca venoms.*

Ciscotto et al. [17] identified that most proteins (12-14 kDa) that were found are similar to PLA_2_ and indicated the presence of both acidic and basic PLA_2_ in *M. frontalis, M. ibiboboca, M. lemniscatus and M. spixii.* In general, basic PLA_2_ enzymes are more toxic and exhibit higher pharmacological potency than their neutral and acidic counterparts, being the basic residues responsible for such potency and lethality [18]. Aside from displaying enzymatic activities, some vPLA_2_ possess a wide range of toxic effects, including neurotoxicity, myotoxicity, cardiotoxicity, cytotoxicity, and may provoke convulsion and hypotension or affect blood coagulation and platelet aggregation [17].

Toxins from Elapid snake venoms play an important role in the characterization and function of mAChRs in muscle and in the identification of muscarinic and nicotinic subtypes of receptors in the central and peripheral nervous system. The venom of Elapid snakes of the genus *Dendroaspis* (mambas) and *Naja* contain 3FTx muscarinic neurotoxins with activity in mAChRs. Moreover, these have a high affinity for a specific receptor subtype. In addition, muscarinic toxins isolated from these venoms with agonist and antagonist features have also been described [19-25]. In this way, we previously characterized the biochemical and pharmacological features of a 3FTx, MT-Mlα, isolated from *Micrurus lemmiscatus* venom. This toxin could displace the binding of the selective muscarinic ligand [^3^H]quinuclidinyl benzilate ([^3^H]QNB) in rat hippocampus. Furthermore, studying pathways of second messengers that can be involved in the effects of the MT-Mlα, our results demonstrated that this toxin inhibited the total [^3^H]inositol phosphate accumulation induced by muscarinic agonist carbachol [26]. 

A new class of muscarinic neurotoxins has also been described. Thus, elapid PLA_2_ neurotoxins isolated from *Naja naja sputatrix* [27, 28] and *Naja atra* [29] venoms have a muscarinic inhibitor activity. In addition, previously studies from our laboratory showed the neurotoxicity of four PLA_2_ (Mlx-8, 9, 11, and 12) isolated from the elapid *Micrurus lemniscatus* snake venom after microinjection into the brain [30]. Those studies showed the presence of isolated and clustered spikes on EEG records. These behavioral alterations were characterized mainly by forelimb clonus, compulsive scratching, and severe neuronal damage. A recent study investigated in detail the neurotoxic effects of two PLA_2_ toxins (Mlx-8 and Mlx-9) isolated from *Micrurus lemniscatus* venom on cultured primary hippocampal neurons. These data demonstrated that the PLA_2_ toxins Mlx-8 and Mlx-9 induce an early increase in free cytosolic calcium concentration and mitochondrial function impairment, which would lead to structural changes and could explain the toxicity to hippocampal neurons. Furthermore, the morphological approaches showed features of hybrid cell death with apoptotic, autophagic, and necrotic signs [15]. Interestingly, a recent isoform of the Mlx-8 toxin named Lemnitoxin has PLA_2_ activity was also isolated from *Micrurus lemniscatus* venom. This was cytotoxic to differentiated myotubes *in vitro* and muscle fibers *in vivo*. A pro-inflammatory activity was also described [31].

We have launched a search for components associated with mAChRs in the venom of the Brazilian snake *Micrurus lemniscatus*. We examined different peaks isolated from this venom (named earlier Mlx-1, Mlx-2, Mlx-3, Mlx-4, Mlx-5, MT-Mlα and Mlx-8). These were obtained from the analytical RP-HPLC profile of *Micrurus lemniscatus* venom on a C8 column. The components were also examined for their ability to compete with [^3^H]QNB for its binding sites. However, only MT-Mlα (a 3FTx; [26]) and Mlx-8 (a PLA_2_-neurotoxin; unpublished data) could displace the binding of the muscarinic ligand. In addition, partial amino acid sequences were determined for MT-Mlα [26] and Mlx-8 (unpublished data). Based on these previous results, the present study investigated the biochemical and pharmacological features of Mlx-8 isolated from *Micrurus lemniscatus* venom with affinity for mAChRs. Thus, this work describes the purification, molecular mass determination, partial amino acid sequencing, and phospholipase A_2_ activity determination of Mlx-8. Furthermore, we characterize its effects on the inhibition of the binding of the selective muscarinic ligand [^3^H]QNB as well as inhibition of the total [^3^H]inositol phosphate accumulation in male rat hippocampus.

## Methods

### Venom

*Micrurus lemniscatus* crude venom was obtained from the Laboratory of Herpetology, Butantan Institute, São Paulo, Brazil. The venom was a pool of several specimens collected in the Southeast region of Brazil. It was lyophilized and stored dry at -20°C until use.

### Animals

The conduct and procedures involving animal experiments were approved by the Butantan Institute Committee for Ethics in Animal Experiments (license number CEUAIB 1100/13) in compliance with the recommendations of the National Council for the Control of Animal Experimentation of Brazil (CONCEA). All efforts were made to minimize animal suffering.

Male Wistar rats (90 day old; 324.8 ± 3.1 g, n = 52), coming from the Central Animal Laboratory of the Butantan Institute, were housed in a polypropylene box (inside length × width × height = 56 cm × 35 cm × 19 cm) (5 animals/cage containing wood shavings) within a ventilated container (Alesco Ind. Com Ltda, Brazil) under controlled temperature (23 ± 2^o^C), relative humidity (65 ± 1%) and 12-h light/12-h dark cycle (lights on at 6:00 a.m.). The animals were allowed to feed and to drink water *ad libitum*.

### Drugs and Radiochemicals

Carbachol (carbamylcholine chloride), lithium chloride, myo-inositol, pirenzepine (pirenzepine hydrochloride), HPLC grade acetonitrile, and trifluoroacetic acid were obtained from Sigma Chemical Co. (USA). The 7,7-dimethyl-5,8-eicosadienoic acid (DEDA) was obtained from Abcam Laboratories (USA). The [^3^H]quinuclidinyl benzylate (specific activity 47 Ci/mmol) was obtained from New England Nuclear (USA). Myo-[1,2-^3^H] inositol (specific activity 18 Ci/mmol) was purchased from Amersham (UK). The OptiPhase HiSafe 3 was obtained from Perkin Elmer (UK). The AG^(^ 1-X8 (200 - 400 mesh) resin was purchased from Bio Rad Laboratories (Richmond, CA, USA). All other drugs and reagents were obtained from Merck (Darmstadt, Germany) or Sigma Chemical Co.

### **RP-HPLC Purification of *Micrurus lemniscatus* Venom**

*Micrurus lemniscatus* crude venom (30 mg) was diluted in 3 mL of Milli-Q water and purified as described by da Silva et al. [26]. Briefly, after filtration in a 0.45-μm filter (Millipore), 800-μL samples (400 μg) were applied to a C8 reversed-phase column (Shim-Pack; 4.6 mm× 250 mm, 5-μm particle) coupled to a HP 1100 series HPLC system. The elution used a flow rate of 1 mL.min^-1^, and this was monitored at 214 nm. The proteins were eluted with a linear gradient of trifluoroacetic acid (TFA) (solvent A) (0.1% TFA in water) and acetonitrile (solvent B) (90% acetonitrile + 10% A) from 10% to 35% of B over 80 min. Thirty-one fractions were manually collected according to their absorbance. Fractions that contained the Mlx-8 were purified in a C18 RP-HPLC column (SUPELCOSIL-LC-18-DB 15 cm × 4.6 mm cat. no. 58348) eluted with a gradient of 0 to 90% acetonitrile (ACN JT Baker) containing 0.1% of TFA. Solvent A was 0.1% TFA (in Milli-Q water), and solvent B was 90% ACN with 0.1% TFA. The purified Mlx-8 toxin was assayed for its ability to inhibit the binding of selective muscarinic ligand [^3^H]QNB. Moreover, the total [^3^H]inositol phosphate was also determined for pharmacological performance. 

### Mass Spectrometry

The samples were mixed in a saturated aqueous solution containing sulfuric acid (1:1 v/v) and synergistic acid (90% of 2,5-dihydroxybenzoic acid and 10% of α-cyano-4-hydroxycarnamic acid) as described by da Silva et al. [26]. Briefly, a cation exchange step was added immediately before the analysis on an AnchorChip 600/384 MTP plates. This was co-crystallized at ambient temperature, and the samples were processed with reagents from Sigma-Aldrich (USA). The α-cyano-4-hydroxycinnamic acid MALDI matrix was processed with Millipore® C18 Ziptips. MALDI-TOF mass spectrometry was performed on an Axima Performance MALDI-TOF/TOF (Shimadzu, Japan) using an α-cyano-4-hydroxycinnamic acid as the matrix. The peptide profile was acquired in linear mode with 75 V laser power. 

### N-terminal Sequence Determination

The purified protein (500 pmol) was dissolved in ACN 37% to determine the N-terminus sequence as described by da Silva et al. [26]. Briefly, this was processed with Edman degradation using a PPSQ-21A Protein Sequencer following the manufacturer’s instructions and protocols (Shimadzu, Japan). The N-terminal sequence was analyzed with the Expert Protein Analysis System (http://www.expasy.org/) and the Blast platform was adopted to perform the sequence search (<https://web.expasy.org/tmp/1week/blastf25027.html>). The sequences alignments were performed with ClustalW (http://www.ebi.ac.uk/clustalw/). 

### Phospholipase A_2_ Activity

The purified Mlx-8 toxin and crude venom were obtained from *Micrurus lemniscatus* and were assayed for phospholipase A_2_ activity using 4-nitro-3 (octanoloxy) benzoic acid (NOBA) as the substrate [32]. Different protein concentrations in 20 µL of 150 mM NaCl were incubated with 20 µL of 3 mM NOBA in acetonitrile and 100 µL of a buffer containing 10 mM Tris, 10 mM CaCl_2_, and 100 mM NaCl, pH 8. Plates were incubated for 30, 40, and 60 min at 37°C, and absorbance was recorded at 425 nm using a Spectra Max 190 plate reader (Molecular Devices, USA) after addition of 20 µL of 2.5% Triton X-100. The results were expressed as mmol/min/mg of protein of one experiment performed in triplicate. 

In another series of experiments, 200 μM DEDA, a PLA_2_ inhibitor, was incubated in the presence of 2.8 μg Mlx-8 for 60 min and the phospholipase A_2_ activity was determined as described above.

### [^3^H]Quinuclidinyl Benzilate ([^3^H]QNB) Binding Assay

The hippocampus membrane was collected from six animals per experiment and was prepared as described previously [33]. Briefly, the hippocampi were isolated from rats, minced, and homogenized in 25 mM Tris-HCl, pH 7.4 (containing 0.3 M sucrose, 5 mM MgCl_2_, 1 mM EDTA, and 1 mM phenylmethylsulfonyl fluoride) with a Ultra-Turrax homogenizer (T-25, Ika Labortechnik, Staufen, Germany). The homogenate was centrifuged at 1,000 × g for 10 min. The supernatant was then filtered through two layers of gauze and centrifuged at 100,000 × g for 60 min. The final 100,000 × g pellet was re-suspended in 1 mL of 25 mM Tris-HCl, pH 7.4 (containing 5 mM MgCl_2_, 1 mM EDTA, and 1 mM phenylmethylsulfonyl fluoride) using a Dounce homogenizer and stored at -80^o^C. All procedures were performed at 4^o^C, and all solutions contained freshly added 1 mM phenylmethylsulfonyl fluoride to inhibit proteolysis. The total protein concentration in the membrane preparations was determined with a protein reagent assay (Bio Rad Laboratories Inc., USA).

Competition binding experiments were performed as previously described [34]. Briefly, the hippocampus membrane solution (80 µg protein/mL) was incubated with [^3^H]QNB (concentration near the K_D_ values) [33] for 1 h at 30^o^C in the absence and presence of increasing concentrations of Mlx-8 toxin or muscarinic antagonist pirenzepine (control). In another series of experiments, 1 μM Mlx-8 toxin was incubated with [^3^H]QNB in the absence or presence of 200 μM DEDA [27, 35, 36] as described above.

Competition binding data were analyzed using a weighted nonlinear least-squares interactive curve-fitting program GraphPad Prism (GraphPad Prism Software Inc, USA). A mathematical model for one or two binding sites was applied. The inhibition constant (K_i_) was determined from competition curves using the Cheng and Prusoff equation [37]. The potency of the antagonist was expressed via the negative logarithm of their K_i_ value (pK_i_). 

### Measurement of Total [^3^H]inositol Phosphate

Hippocampi were isolated from rats and washed with a nutrient solution of the following composition (mM): NaCl 118.00; KCl 4.78; CaCl_2_ 2.43; MgSO_4_ 1.16; NaHCO_3_ 23.80; KH_2_PO_4_ 1.17; and glucose 2.92 (pH 7.4). Hippocampus slices (100 mg of tissue) were allowed to equilibrate for 10 min in nutrient solution at 37^o^C under constant shaking. The slices were incubated for 40 min with 1 µCi myo-[^3^H]inositol and for an additional 30 min with 10 mM lithium chloride with myo-[^3^H]inositol. Tissues were then incubated in the absence (basal level) or presence of carbachol (CCh, 10^-8^ to 10^-3^ M) for 40 min. Mlx-8 toxin (10^-7^ and 10^-6^ M) was added 5 min prior to incubation with CCh (10^-5^ M). Tissues were washed three times with nutrient solution, transferred to 2 mL of methanol:chloroform (2:1 v/v) at 4^o^C, and homogenized with a Ultra-Turrax T25 homogenizer at 9,500 rpm. Chloroform (0.62 mL) and H_2_O (0.93 mL) were added to the homogenate, and the solution was centrifuged for 10 min at 2,000 x g and 4^o^C to separate the aqueous and organic phases [38, 39].

Total [^3^H]inositol phosphate was measured as previously described [40] with the following modification: the aqueous layer was mixed with 1 mL anion-exchange resin (Dowex AG-X8, formate form, 200-400 mesh) allowed to equilibrate for 30 min at room temperature. It was then centrifuged at 1,000 × g for 5 min at 4^o^C. The resin was sequentially washed with myo-inositol (4 mL) and 5 mM sodium tetraborate/60 mM sodium formate (2 mL). Subsequently, the resin was incubated for 30 min at room temperature with 2 mL of 0.1M formic acid/1M ammonium formate. The total [^3^H]inositol phosphate was eluted and placed in scintillation vials containing OptiPhase HiSafe 3. The amount of radioactivity was determined in a scintillation β-counter (LS 6500 IC, Beckman). Total [^3^H]inositol phosphate was expressed as dpm/mg tissue.

### Statistical Analysis

Data were expressed as the mean ± S.E.M. Data were analyzed by ANOVA followed by Newman-Keuls test for multiple comparisons or via a two-tailed Student’s *t*-test to compare a response between the two groups [41]. P values < 0.05 were considered to be significant. 

## Results

### Biochemical Characterization of the Mlx-8 Toxin

**Figure 1:**
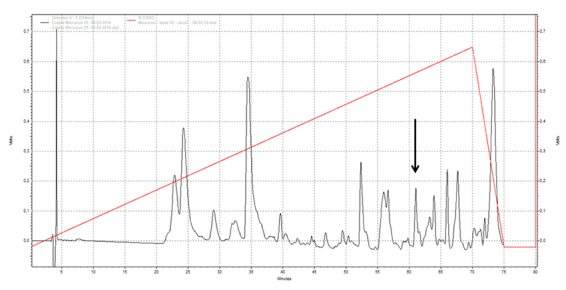
Crude venom purified on high performance liquid chromatography (HPLC) using a C8 column on a Prominence binary system (Shimadzu). The venom components were eluted with a flow rate of 1 mL/min with solvents A [0.1% trifluoroacetic acid (TFA) in deionized H_2_O] and B (90% acetonitrile, 0.1% TFA in deionized H_2_O) with gradient from 10 to 35% of solution B, represented by the trace. The absorbance was read at 214 nm. In the highlighted area, the arrow indicates the fraction that contains the Mlx-8 with a retention time of 61 minutes in 75 minutes run.

**Figure 2:**
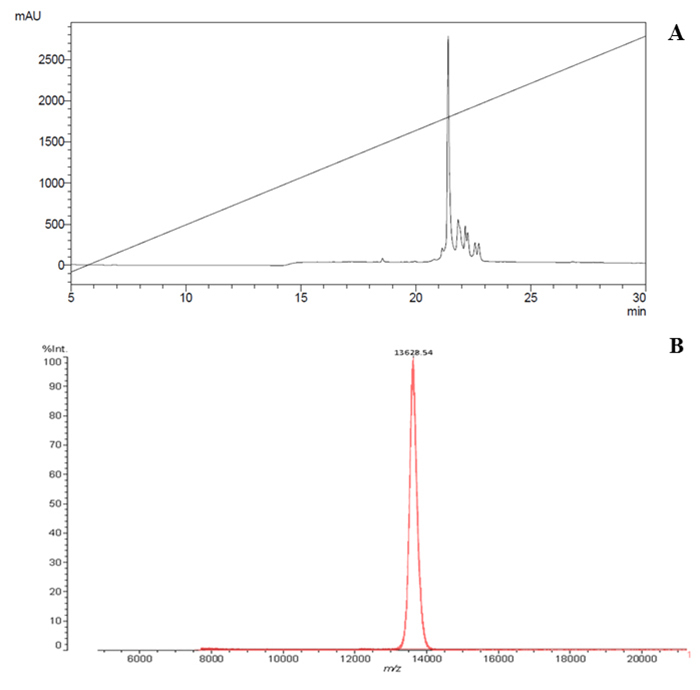
(**A**) The fraction that contains the Mlx-8 was purified on HPLC-RF using a C18 column eluted under a flow rate of 1 mL/min with solvents A and B from 0 to 100% acetonitrile in 0.1% TFA aqueous solution represented by the trace. The absorbance was read at 214 nm. Only the highest peak was collected, thus removing the contaminants from the sample and guaranteeing its purity. (**B**) Mass spectrum of the Mlx-8 toxin obtained via mass spectrometry technique in MALDI-TOF ionization mode. The toxin was analyzed using the saturated sinapinic acid matrix solution (1:1 v/v) and deposited directly onto MCP AnchorChip 600/384 plates. This was co-crystallized at room temperature. After ionization, the Mlx-8 toxin molecules were transformed into ions and counted by the detector as a function of their mass/charge (m/z) and their molecular mass was identified.

Figure 1 presents the RP-HPLC profile of the *Micrurus lemniscatus* venom. The fraction that contains the Mlx-8 was purified in a C18 RP-HPLC column (Fig. 2A): 180 µg of Mlx-8 toxin was obtained from 30 mg of the crude venom. Mlx-8 toxin was collected and had its molecular mass verified by MALDI-TOF. The MS profile was 13,628 as shown for the peak in Figure 2B. 

**Figure 3:**
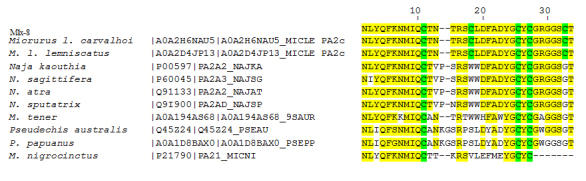
Amino acid multiple sequence alignment. The Mlx-8 toxin obtained from *Micrurus lemniscatus* venom was aligned with other PLA2 from *Micrurus lemniscatus carvalhoi* [9] and from Lemnitoxin from *Micrurus lemniscatus* [31]. In addition, this sequence was analyzed against the public protein data bank to check for similarities with known proteins. Besides that, matches were identified with toxins from *Naja Kaouthia*, *N. sagittifera*, *N. atra*, *N. sputatrix*, *M. tener*, *Pseudechis australis*, *P. papuanus* and *M. nigrocintus venoms*.

The Mlx-8 N-terminal sequence was determined by Edman degradation and the following sequence: NLYQFKNMIQCTNTRSWLDFADYGCYCGRGGSGT (Fig. 3) was obtained. The sequence determination showed that the Mlx-8 presents high similarity to other toxins from Elapidae such as the PLA_2_ from *Micrurus lemniscatus carvalhoi* [9] and from Lemnitoxin from *Micrurus lemniscatus* [31]. In addition, this sequence was analyzed against the public protein data bank to check for similarities with known proteins. Besides that, matches were identified with toxins from *Naja kaouthia*, *N. sagittifera*, *N. atra*, *N. sputatrix*, *M. tener, Pseudechis australis, P. papuanus* and *M. nigrocintus* venoms (Fig. 3).

### Phospolipase A_2_ Activity of the Mlx-8 Toxin

The phospholipase A_2_ enzymatic activity of 2.8 μg Mlx-8 toxin was determined at different time periods (30, 40 and 60 minutes). The Mlx-8 could hydrolyze the substrate to phospholipase A_2_, 4-NOBA at all times. The 60-minute time point revealed a greater activity (229.4 ± 14.33, 345.5 ± 9.12, and 582.8 ± 29.3 mmol/min/mg, respectively, 30, 40 and 60 minutes). 

The amount of 2.8 μg Mlx-8 in the presence of 200 μM DEDA for 60 min decreased 51% (285.57 ± 7.47 mmol/min/mg) the phospholipase A_2_ enzymatic activity. 

### Effect of Mlx-8 Toxin on [^3^H]QNB Binding in Hippocampus Membranes

**Figure 4:**
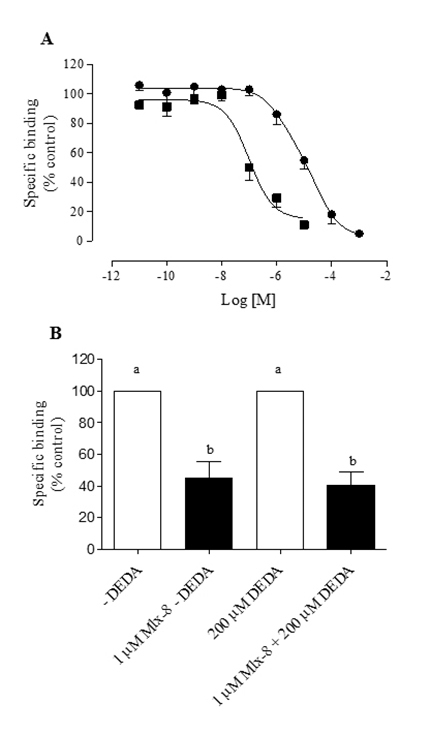
(**A**) Displacement curves of [^3^H]QNB bound to hippocampus membranes from male rats induced by Mlx-8 toxin (⯀) and muscarinic acetylcholine receptor antagonist pirezenpine (●). (**B**) Displacement of [^3^H]QNB bound to hippocampus membrane by 1 µM Mlx-8 toxin obtained from *Micrurus lemniscatus* venom in the absence and presence of 200 µM DEDA, an inhibitor of phospholipase A2. The data are plotted as percentages of the binding in the absence of Mlx-8 or muscarinic acetylcholine receptor antagonists. Each point and vertical line represents the mean ± S.E.M. of n = 4, performed in duplicate. Different letters indicate statistical significance (p < 0.05; ANOVA, Newman–Keuls test).

Figure 4A shows the displacement curves of [^3^H]QNB bound to hippocampus membranes induced by Mlx-8 toxin and pirezenpine (M_1_ selective antagonist) [42]. Analysis of the displacement curves induced by Mlx-8 toxin and pirenzepine indicated a statistical preference for a one-site rather than a two-site fit (*F*-test, GraphPad Prism program). The pK_i_ values obtained from the analysis of Mlx-8 and antagonist displacement curves via one-site fit and their respective Hill slopes (n_H_) were 7.32 ± 0.15, n = 4 (n_H_ = 1.14 ± 0.13) and 5.84 ± 0.18, n = 4 (n_H_ = 0.94 ± 0.15) for Mlx-8 and pirenzepine, respectively.

 The 200 µM DEDA had no effect on the inhibition of [^3^H]QNB binding in hippocampus membranes when using 1 µM Mlx-8 (Fig. 4B).

### Effects of Carbachol and Mlx-8 Toxin on total [^3^H]inositol Phosphate Accumulation

**Figure 5:**
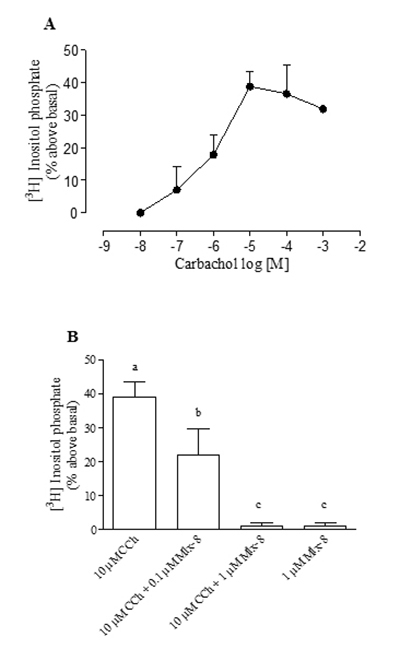
(**A**) Concentration-effect curve of carbachol (CCh) on total [^3^H]inositol phosphate accumulation. (**B**) Effect of the Mlx-8 toxin on total [^3^H]inositol phosphate accumulation induced by 10^-5^ M CCh in the hippocampi from male rats. Each point and vertical line represent the mean ± S.E.M. of n = 3. Different letters indicate statistical significance (p < 0.05; ANOVA, Newman-Keuls test).

The basal level of the total [^3^H]inositol phosphate in rat hippocampus was 69.12 ± 5.80 dpm/mg tissue, n = 12. The cholinergic agonist carbachol (CCh, 10^-8^ M to 10^-3^ M) caused a concentration-dependent increase in the hippocampal total [^3^H]inositol phosphate accumulation (Fig. 5A). The maximum total [^3^H]inositol phosphate accumulation was obtained with 10^-5^ M CCh (38.80 ± 4.50% above basal, n = 4) (Fig. 5A).

The purified Mlx-8 toxin (10^-7^ and 10^-6^ M) obtained from *Micrurus lemniscatus* snake venom did alter the total [^3^H]inositol phosphate accumulation induced by 10^-5^ M CCh in a concentration-dependent manner. The Mlx-8 toxin caused a blockade of 43.31 ± 8.86% (n = 3) and 97.42 ± 2.02% (n = 3) at 10^-7^ and 10^-6^ M, respectively, on [^3^H]inositol phosphate accumulation induced by 10^-5^ M CCh in the rat hippocampus (Fig. 5B). In the absence of CCh, the Mlx-8 toxin (10^-6^ M) did not alter the total [^3^H]inositol phosphate accumulation in the hippocampus (Fig. 5B) (p < 0.05; ANOVA, Newman-Keuls test).

## Discussion

The results show for the first time that the mAChRs function is drastically affected by Mlx-8 toxin, a muscarinic ligand with phospholipase A_2_ activity obtained from *Micrurus lemniscatus* venom. This species is in the Elapidae family, and its toxin can inhibit binding of the selective muscarinic ligand [^3^H]QNB in rat membranes from the hippocampus. Furthermore, the toxin also inhibited [^3^H]inositol phosphate accumulation in the hippocampus. 

Muscarinic toxins that affect ligand binding to mAChR have been isolated from mamba venom [see 43-45, for review]. The structures of this group of toxins are somewhat similar to the postsynaptic neurotoxins and consist of three polypeptide loops (3FTx). They all share roughly the same number of amino acids (63-66 AA) and molecular weight (about 7 kDa). However, the molecular mass of Mlx-8 (13.6 kDa) from the venom of *Micrurus lemniscatus* seen here is clearly different from muscarinic toxins. In this way, a similar molecular mass of Mlx-8 was observed versus muscarinic toxins with phospholipase A_2_ activity obtained from *Naja naja sputatrix* (13.6 kDa) [27] and *Naja atra* (13.3 kDa) [29]. This indicates that Mlx-8 may belong to a group of snake PLA_2_-toxins. 

Indeed, when N-terminal analysis and alignment of Mlx-8 (NLYQFKNMIQCTNTRSWL-DFADYG-CYCGRGGSGT) was determined and compared to other proteins with muscarinic activity, the data revealed a high similarity to Elapidae venom proteins including a neural phospholipase A_2_ muscarinic inhibitor from *Naja naja sputatrix* (NLYQFKNMIQCTVPNR) [27] and *Naja atra* (NLYQFKNMIQCTVPSR) [29]. Recently, a toxin named Lemnitoxin was isolated from *Micrurus lemniscatus* venom and shown to be a PLA_2_ with myotoxic and pro-inflammatory activity [31]. The N-terminal comparison of the Mlx-8 toxin with Lemnitoxin (NLYQFKNMIQCTNTRSWL-DFADYG-CYCGYGGSGT) revealed an almost identical amino acid sequence between both toxins suggesting either a very similar toxin or an isoform. Other studies are needed to prove this issue. The Mlx-8 toxin was strongly expected to have phospholipase A_2_ activity in view of the biochemical properties described above. In fact, Mlx-8 shows phospholipase A_2_ enzymatic activity. 

The mAChRs mediate a wide range of functions of the parasympathetic nervous system both centrally and peripherally. Different experimental approaches have shown that mAChRs are present in all organs, tissues, or cell types [see 46, for review]. The muscarinic actions of acetylcholine are mediated by five distinct mAChR subtypes (M_1_ to M_5_) [47-49]. The M_1_, M_3_, and M_5_ subtypes couple primarily to phospholipase C-mediated phosphoinositide hydrolysis. On the other hand, the M_2_ and M_4_ subtypes couple primarily to adenylyl cyclase inhibition [see 50, for review]. To characterize the effect of Mlx-8 toxin on mAChRs at the protein level, Mlx-8 and the M_1_ selective muscarinic antagonist pirenzepine were examined for their ability to compete with [^3^H]QNB for binding sites in the hippocampus membrane. The pK_i_ of the Mlx-8 (7.32) was higher than that obtained by pirenzepine (5.84). Moreover, the Hill slope coefficients calculated for Mlx-8 and pirenzepine did not differ from unity. These data support the idea that Mlx-8 has affinity for mAChRs. Further experimental approaches are needed to clarify the mechanisms involved and the functional significance of Mlx-8 on mAChRs. 

This study focused only on the phospholipase C-mediated phosphoinositide hydrolysis in hippocampal tissue because the population of M_1_ receptors is predominant in the rat hippocampus [see 46, for review]. The Mlx-8 toxin obtained from *Micrurus lemniscatus* venom reduced the response to carbachol on total [^3^H]inositol phosphate accumulation in a concentration-dependent manner. In the absence of carbachol, 1 µM Mlx-8 did not alter the level of total [^3^H]inositol phosphate. These studies collectively indicate that the Mlx-8 toxin blocked the intracellular signaling pathway linked to activation of mAChRs in rat hippocampus. Interestingly, the Mlx-8 toxin is quite different from the toxin obtained from *Naja atra* venom [29]. Although both exhibit similarity of the N-terminal amino acid sequence and molecular mass, Mlx-8 (1 µM) inhibits the total [^3^H]inositol phosphate accumulation (97%) induced by muscarinic agonist carbachol while the *Naja atra* venom promotes contraction in the ileum of guinea pig via mAChRs [29]. Whether the Mlx-8 toxin plays a role in other intracellular signaling pathways coupled to mAChRs remains to be explored.

Specific binding membrane receptor proteins of venom phospholipase A_2_ have been shown. For example, vipoxin (a minor PLA_2_ from *Vipera russelli* venom) can bind to amine receptors on rat brain [51]. OS2 is a single-chain PLA_2_ isolated from *Oxyuranus scutellatus* venom and associates selectively with rat brain membrane proteins termed N-type receptors [52]. Moreover, there is evidence suggesting that the ability to interact with nicotinic acetylcholine receptors may be a general property of several snakes PLA_2_ from venoms [53, 54]. To check the ability of the PLA_2_ isolated from *Micrurus lemniscatus* (Mlx-8) to interact with mAChRs, the inhibitor of cobra venom phospholipases A_2_ activity DEDA, an analogue of arachidonic acid that contains two cis double bonds as well as two methyl groups [55], was used in the present study. Indeed, the phospholipase A_2_ enzymatic activity of Mlx-8 in the presence of DEDA decreased 51%. Interestingly, there was no impact on inhibition of [^3^H]QNB binding in hippocampus membranes via DEDA, suggesting that the inhibition of the phospholipase A_2_ activity of the venom did not alter its ability to bind and displace [^3^H]QNB binding. Similarly, DEDA did not also block the mAChRs binding in muscarinic toxin with PLA_2_ activity obtained from *Naja naja sputatrix* venom [27]. On the other hand, the inhibitor of phospholipases A_2_ activity p-bromophenacyl bromide, which modifies the histidine residue in the active site of PLA_2_, eliminated both PLA_2_ activity and [^3^H]QNB binding [27]. Thus, only DEDA showed no effect on mAChRs binding when used [28].

*Micrurus* venoms are natural libraries of biologically active molecules that can be used as new drug leads. However, a major obstacle to characterize the components of *Micrurus* venoms is the minute quantities of material obtained from specimen milking. Thus, despite the large variety of molecules with potential biotechnological application, there is still a great difficulty of their bioprospecting due to the small amount of starting material, low yield and the high cost of traditional purification strategies. In general, this alone explains the small number of animal molecules currently used as drugs. The recent development and use of “omic” tools has become increasingly prominent since they allow an overview of the composition of the venom. In addition, the transcriptome technique, associated with the cloning and heterologous expression of proteins and peptides, enables the production of molecules present in the gland or specialized tissue in sufficient quantity for their structural and functional analysis. Therefore, these studies enable the application of molecules with relevant biological activity. From this perspective, as regards Mlx-8, further studies will be required to better explore the biological potential of this toxin.

## Conclusion

The results of the present work show, for the first time, that mAChRs are also affected by the Mlx-8 toxin, a muscarinic ligand with phospholipase A_2_ characteristics, obtained from the venom of the Elapidae snake *Micrurus lemniscatus*, since this toxin was able to compete with muscarinic ligand [^3^H]QNB in hippocampus from ​ rats. In addition, Mlx-8 also blocked the accumulation of total [^3^H]inositol phosphate induced by muscarinic agonist carbachol. Thus, Mlx-8 may be a new pharmacological tool for examining muscarinic cholinergic function.

### Abbreviations

 [^3^H]QNB: [^3^H]quinuclidinyl benzilate; 3FTx: three finger; CCh: carbachol; DEDA: 7,7-dimethyl-5,8-eicosadienoic acid; HPLC: high performance liquid chromatography; K_i_: inhibition constant; mAChRs: muscarinic acetylcholine receptors; Mlx-8 and MT-Mlα: toxins isolated from *Micrurus lemniscatus* venom; NOBA: 4-nitro-3 (octanoloxy) benzoic acid; PLA_2_: phospholipase A_2_; TFA: trifluoroacetic acid.
